# Variation in guideline adherence in non-Hodgkin’s lymphoma care: impact of patient and hospital characteristics

**DOI:** 10.1186/s12885-015-1547-8

**Published:** 2015-08-08

**Authors:** Jozette J.C. Stienen, Rosella P.M.G. Hermens, Lianne Wennekes, Saskia A.M. van de Schans, Richard W.M. van der Maazen, Helena M. Dekker, Janine Liefers, Johan H.J.M. van Krieken, Nicole M.A. Blijlevens, Petronella B. Ottevanger, On behalf of the PEARL study group

**Affiliations:** 1 Scientific Institute for Quality of Healthcare (IQ healthcare), Radboud university medical center (Radboud umc), PO Box 9101, 6500 HB Nijmegen, the Netherlands; 2 Department of Radiotherapy, Radboud university medical center, Nijmegen, the Netherlands; 3 Department of Radiology, Radboud university medical center, Nijmegen, the Netherlands; 4 Department of Pathology, Radboud university medical center, Nijmegen, the Netherlands; 5 Department of Hematology, Radboud university medical center, Nijmegen, The Netherlands; 6 Department of Medical Oncology, Radboud university medical center, Nijmegen, the Netherlands; 7 Netherlands Comprehensive Cancer Organisation, Department of Registry and Research, PO box 19079, 3501 DB Utrecht, the Netherlands

**Keywords:** Non-Hodgkin’s lymphoma, Hematology, Guidelines, Determinants, Hospital variation, Oncology

## Abstract

**Background:**

The objective of this observational study was to assess the influence of patient, tumor, professional and hospital related characteristics on hospital variation concerning guideline adherence in non-Hodgkin’s lymphoma (NHL) care.

**Methods:**

Validated, guideline-based quality indicators (QIs) were used as a tool to assess guideline adherence for NHL care. Multilevel logistic regression analyses were used to calculate variation between hospitals and to identify characteristics explaining this variation. Data for the QIs regarding diagnostics, therapy, follow-up and organization of care, together with patient, tumor and professional related characteristics were retrospectively collected from medical records; hospital characteristics were derived from questionnaires and publically available data.

**Results:**

Data of 423 patients diagnosed with NHL between October 2010 and December 2011 were analyzed. Guideline adherence, as measured with the QIs, varied considerably between the 19 hospitals: >20 % variation was identified in all 20 QIs and high variation between the hospitals (>50 %) was seen in 12 QIs, most frequently in the treatment and follow-up domain.

Hospital variation in NHL care was associated more than once with the characteristics age, extranodal involvement, multidisciplinary consultation, tumor type, tumor aggressiveness, LDH level, therapy used, hospital region and availability of a PET-scanner.

**Conclusion:**

Fifteen characteristics identified at the patient level and at the hospital level could partly explain hospital variation in guideline adherence for NHL care. Particularly age was an important determinant: elderly were less likely to receive care as measured in the QIs. The identification of determinants can be used to improve the quality of NHL care, for example, for standardizing multidisciplinary consultations in daily practice.

## Background

Non-Hodgkin’s lymphoma (NHL) is the most common hematologic neoplasm worldwide, and affects over 300,000 people each year [1]. In the United States, NHL is the sixth most common cancer with an estimated number of almost 70,000 new cases in 2013 [2]. This heterogeneous group of malignant proliferations of lymphocytes consists of more than 40 disease entities. Approximately 50 % of the cases comprises the types diffuse large B-cell lymphoma (DLBCL) and follicular lymphoma [3].

Treatment of NHL is highly dependent on the type and stage of the tumor. Primary therapy options include chemotherapy, radiation therapy, immunotherapy and wait-and-see policy. More effective therapy options are emerging, partly due to many randomized controlled trials in this field. Despite these improvements, the five-year relative survival rate is still rather low for DLBCL patients (55–60 %), and for patients diagnosed with follicular lymphoma this is 74–86 % [4, 5].

The emerging diagnostic and therapy options require evidence-based guidelines to assist professionals and patients in their decision-making process for NHL care. These guidelines should be in line with the description of care of the Institute of Medicine (IOM): care should be safe, effective, patient-centered, timely, efficient and equitable [6]. However, previous studies showed variation in care for NHL patients based on discrepancies between daily practice and recommendations in guidelines [7–9]. Actual guideline adherence was assessed with quality indicators, defined as ‘measurable elements of practice performance for which there is evidence or consensus that they can assess the quality of the care provided’ [10]. The suboptimal adherence to guidelines in NHL patient management can be an indication of suboptimal quality of care and therefore may require tailored interventions, since quality of care does not improve by itself. In order to develop tailored improvement strategies, it is important to gain more insight into factors that influence guideline adherence in daily practice on patient and hospital level. In previous studies, determinants of NHL care focused on patient and tumor characteristics, such as age, tumor stage and co-morbidity score [7–9, 11, 12]. However, little is known about the possible influence of hospital factors. In other healthcare settings, patients’ age and diagnosis are often associated with guideline adherence [13–15], however, hospital factors (e.g. hospital size) seem important to consider as well [13, 16–19].

In the current study, we assessed hospital variation in guideline adherence in NHL care and to what extent these variations can be explained by differences on patient and hospital level. This report builds upon previous work where quality indicators were developed and measured to provide insight into guideline adherence for NHL care [9, 20]. Together with insight into variation in guideline adherence and accompanying determinants, tailored strategies to improve NHL care can be designed.

## Methods

### Study design and population

This observational study was performed using baseline measurements of the PEARL study (improvement of patients’ hospital care for non-Hodgkin’s lymphoma), a cluster randomized controlled trial (cRCT) to test and evaluate tailored strategies to improve hospital care for patients with NHL (registered at ClinicalTrial.gov: NCT01562509) [21].

The extent of hospital variation was assessed in 19 hospitals across three regions of the Netherlands (north, east and south), including university, teaching and non-teaching hospitals. Patients eligible for this study were defined as patients diagnosed with a mature B-, T- or NK-cell neoplasm between October 2010 and December 2011, and older than 18 years at diagnosis. Patients with cutaneous lymphomas or leukemia-type neoplasms were excluded. The Dutch cancer registry was used by the Netherlands Comprehensive Cancer Organisation (IKNL) to make a list of potentially eligible patients in the participating hospitals. For each hospital a random sample of 25–30 patients was selected for data collection.

### Data collection

#### Quality indicators

Data were assessed using systematically developed and validated quality indicators (QIs), based on (inter)national evidence-based guidelines [9]. This set of 20 QIs was developed by professionals involved in NHL care and covers important processes and structures in management of NHL care in the domains diagnosis and staging, treatment and follow-up, and organization and coordination of care. In short, the QIs reflect quality of NHL care as described in guidelines. Trained registration employees, from the IKNL, collected data from medical records for the QIs using predefined registration forms. Room for improvement was defined if quality indicator scores were less than 90 % [9, 22–24].

#### Patient, tumor, professional and hospital related characteristics

The characteristics were selected because of their potential association with guideline adherence and quality of NHL care, based on prior research findings [9, 11, 12, 25]. Potentially relevant patient and tumor related characteristics were age (continuous), gender (male/female), co-morbidities (yes/no), performance status (good/bad, good indicating a WHO score <2 or Karnofsky score ≥60), patients’ preferences (yes/no objections), previous malignancies (yes/no), tumor aggressiveness (yes/no), extranodal involvement (yes/no, this term is used if the disease is not in the lymph nodes (extranodal) or has spread from lymph nodes to extranodal sites), Ann Arbor disease stage (I/II or III/IV), tumor type (yes/no DLBCL), International Prognostic Index (IPI) score (low/intermediate/high), lactate-dehydrogenase level (LDH, yes/no high level (>250 u/L)) and hemoglobin level (Hb, yes/no aberrant level (<7.5/8.5 or >10/11, females/males)). Factors related to professionals (dichotomous, yes/no) included multidisciplinary team consultation (MTC), discussion in pathology panel, in-hospital referral and therapy used (watch-and-wait was defined as ‘no therapy used’). Patient, tumor and professional related factors were all collected from medical records at patient level.

The hospital characteristics include type of hospital (yes/no teaching hospital), region of hospital (north/east/south), availability of an in-hospital pathology laboratory (yes/no) and PET-scanner (yes/no) and availability of professionals specialized in hematology, including a specialized oncology nurse (yes/no). Hospital characteristics were collected for each hospital from publically available data as well as from a short, digital questionnaire (multiple choice questions) sent to the contact person (oncologist or hematologist) at each hospital.

### Statistical analysis

Quality indicator scores and hospital variation were calculated to provide insight into guideline adherence in NHL care, which gives an indication of the quality of care as delivered to NHL patients. Patient, tumor, professional and hospital related characteristics were described by calculating frequencies and means. Univariate analyses (*X*^2^-test and *t*-test) were performed to study correlations between the QI scores (dependent variables) and the selected characteristics (independent variables). Single correlations were only tested for QIs and characteristics if the link between the two factors is clinically explicable (e.g. radiology related QIs were not tested for pathology related characteristics, since these processes are independently performed from each other).

Multivariate logistic regression was performed to study correlations for those characteristics with *P* < 0.20 in univariate analyses. Correlations between the independent variables were also tested. If a correlation (>0.8) was detected, only one variable was included in the multivariate analyses.

Finally, multilevel logistic regression analysis was used to determine to what extent the QI scores were influenced by the characteristics [26]. Multivariate backwards regression models, including random coefficients, were constructed for each quality indicator. The reason for using this analysis was the hierarchical nature of the characteristics, as patients (level 1) were nested in hospitals (level 2). We considered *P* < 0.05 as statistically significant and calculated the explained variance (R^2^) per multilevel model with the Glimmix procedure using SAS software (SAS12.0 for Windows; SAS Institute, Cary, North Carolina, USA). Odds ratios (OR) were used to describe the association between the characteristics and quality indicator. An OR >1 indicates a positive association with the quality indicator (greater relative chance of guideline adherence if the determinant is present).

### Ethics

On behalf of the research ethics committee (CMO) of the Radboud university medical center, we hereby let you know that the current study has been carried out in accordance with the applicable rules concerning the review of research ethics committees and informed consent (registration number 2011/560).

The IKNL has contracts with each Dutch Hospital about the Cancer Registry that all patients are informed about the registration and are registered unless the patient has objected to be registered. The Netherlands Cancer Registry is obliged to work according to the law about protection of privacy data and the law “Geneeskundige BehandelOvereenkomst”. All procedures to privacy of doctors and patients is fixed in regulations. An independent Committee of Privacy reassures that the Netherlands Cancer Registry works is compliant to these regulations. Based on this, consent of the patients for this specific study was not applicable; according to the Dutch law all cancer patients are included in the Netherlands Cancer Registry as maintained by the IKNL, unless the patient has objected to be registered.

## Results

### Patient, tumor and professional related characteristics

Table 1 shows the patient, tumor and professional related characteristics included in this study, measured at patient level. In total, data were collected for 423 patients diagnosed with NHL between 2010–2011 across 19 Dutch hospitals. The mean age of the patients was 66 years (range 22–94), 57 % was male and 61 % had at least one co-morbidity. Tumor related characteristics showed that 61 % had extranodal involvement, 60 % was diagnosed with an aggressive tumor and Ann Arbor stage III of IV was observed in 68 % of the patients. Professional related factors as discussion in a pathology panel and an MTC were performed in 33 and 41 % of the patients, respectively. Of the 423 patients in this study, 75 % received therapy, either chemotherapy, radiotherapy or a combination of these two, as initial treatment. Three factors were excluded from further analyses: performance status and IPI score because of too many missing values (respectively, 83 and 58 % missings) and patient preferences because of <10 % variation (only 6 % had objections concerning diagnostics or therapy).

**Table 1:** Patient related characteristics

Characteristics	Patients	(*N*_*TOTAL*_ = 423)
*Patient factors*	*N*	*%*
Male sex	242	57
Mean age, years (range)	423	66 (22–94)
Co-morbidities (≥1)	256	61
Objections (patient preferences)^A^	25	6
Good performance status^B^	67	94
*Tumor related factors*		
Previous malignancies	71	17
Extranodal involvement	258	61
High LDH level^C^	171	44
Aberrant Hb level^D^	173	42
Ann Arbor stage III/IV	268	68
Aggressive tumor	254	60
DLBCL tumor type^E^	194	46
IPI score (intermediate-) high^B^	49	38
*Professional related factors*		
In-hospital referral	270	64
Multidisciplinary team consultation	172	41
Discussed in pathology panel	137	33
Therapy used^F^	319	75

*Abbreviations*: *LDH* lactate-dehydrogenase, *Hb* Hemoglobin, *DLBCL* diffuse large B-cell lymphoma, *IPI* International Prognostic Index

^A^Excluded from further analyses due to <10 % variation

^B^Excluded from further analyses due to >50 % missings, based on

WHO and Karnofsky scores

^C^High LDH level is defined as >250 U/l

^D^Aberrant Hb level is defined as <8.5 or >11 mmol/l for males and<7.5 or >10 mmol/l for females

^E^Non-DLBCL tumor types include follicular lymphoma (18 %),

marginal zone B-cell lymphoma (11 %), mantle-cell lymphoma (5 %),

lympho(plasma)cytic lymphoma (10 %), and miscellaneous (9 %)

^F^The watch-and-wait management was coded as ‘no therapy used’

### Hospital characteristics

Table 2 outlines the characteristics of the participating hospitals, measured at hospital level. Of the 19 hospitals, 47 % was situated in the Northern region and 53 % comprised teaching hospitals. An in-hospital pathology laboratory was present in 47 % of the hospitals and 42 % had the availability of an in-hospital PET-scanner. Most hospitals (87 %) had a specialized pathologist, whereas 78 % had a specialized oncologist or hematologist and 47 % a specialized radiologist/nuclear physician. In 68 % of the hospitals a specialized oncology nurse was available. Two factors were excluded from further analyses because of high correlation with at least one other hospital characteristic: trial participation and hospital size.

**Table 2:** Hospital characteristics

Characteristics	Hospitals	(*N*_*TOTAL*_ = 19)
	*N*	*%*
Hospital region		
North	9	47
East	5	26
South	5	26
Teaching hospital	10	53
In-hospital pathology laboratory	9	47
In-hospital PET-scanner	8	42
Specialized oncologist or hematologist	14	78
Specialized radiologist/nuclear physician	9	47
Specialized pathologist	16	87
Specialized oncology nurse	13	68
Trial participation^A^	12	63
Hospital size (no. of beds)^A^		
Small (<350)	8	42
Medium (350–650)	5	26
Large (>650)	6	32

^A^Excluded from further analyses due to high correlation with other

hospital characteristics

### Quality indicators

Guideline adherence in NHL care was measured with a set of 20 QIs, presented in Table 3. Room for improvement (<90 % adherence) was seen in 18 out of 20 QIs. Regarding diagnosis and staging, the indicator with the lowest score was performance of all staging techniques (QI4, 48 %) and the indicator with the highest score was diagnosis based on morphology and immune phenotype (QI3, 96 %). Indicators for treatment and follow-up showed a range of 62–82 %, including complete evaluation after chemotherapy (QI11) as lowest score and reporting dose reduction for chemotherapy RCHOP (QI13) as highest score. In the domain of organization and coordination, the lowest score was for complete pathology reports (QI16, 14 %) and the highest score for integrated reporting of pathology techniques (QI15, 89 %).

**Table 3:** Indicator scores and determinants for guideline adherence concerning NHL care

Quality Indicator	N	Indicator score (%)	Range in 19 hospitals(%)	OR (95 % CI)	*P*-value	Explained variance (%)
*Diagnosis and staging*						
QI1 Diagnosis based on histological examination or an excision or wide incision biopsy	369	79	53–100			5
Older age				0.97 (0.95–0.99)	<0.01	
QI2 Patients staged according to Ann Arbor classification	390	81	59–100			35
Older age				0.93 (0.90–0.95)	<0.01	
Therapy used				7.55 (4.04–14.00)	<0.01	
QI3 Diagnosis based on morphology and immune phenotype	376	96	74–100			n.a.
QI4 Staging techniques include CT-scans, bone marrow aspirate, and bone biopsy	421	48	0–74			14
Older age				0.98 (0.96–0.99)	0.02	
Extranodal involvement				0.52 (0.33–0.84)	<0.01	
Discussed in MTC				1.88 (1.10–3.20)	0.02	
Aberrant Hb level				0.54 (0.34–0.86)	0.01	
Therapy used				3.08 (1.76–5.39)	<0.01	
QI5 Assessment of International Prognostic Index for patients with aggressive NHL	250	43	0–81			17
Older age				0.97 (0.95–0.99)	0.01	
DLBCL tumor type				2.85 (1.07–4.82)	0.03	
Therapy used				8.70 (1.82–41.50)	<0.01	
QI6 Assessment of LDH level	423	92	70–100			9
Discussed in MTC				0.42 (0.19–0.94)	0.03	
Therapy used				2.92 (1.36–6.27)	<0.01	
QI7 Examination of blood counts	422	82	14–100			n.a.
*Treatment and follow-up*						
QI8 Reporting of response to therapy using predefined terminology	304	73	53–100			2
High LDH level				1.79 (1.03–3.11)	0.04	
QI9 Lesions documented in radiology report before therapy	344	67	22–90			n.a.
QI10 Lesions documented in radiology report after therapy	114	58	0–100			24
DLBCL tumor type				0.34 (0.15–0.79)	0.01	
Co-morbidities (≥1)				0.34 (0.15–0.81)	0.02	
Hospital region 1				1.08 (0.38–3.07)		
2				4.10 (1.48–11.40)		
3				Ref.	0.02	
QI11 Evaluation after chemotherapy with (PET)CT-scans, bone marrow aspirate, and bone biopsy	246	62	29–100			30
Extranodal involvement				0.18 (0.09–0.34)	<0.01	
Hospital region 1				0.93 (0.47–1.84)		
2				4.54 (1.88–10.96)		
3				Ref.	<0.01	
QI12 Patients with DLBCL received RCHOP chemotherapy	194	78	44–100			26
Older age				0.92 (0.88–0.95)	<0.01	
QI13 Dose of RCHOP was not reduced or reason for reduction was reported	111	82	40–100			8
Extranodal involvement				0.31 (0.10–0.99)	0.05	
Quality Indicator	N	Indicator score (%)	Range in 19 hospitals (%)	OR (95 % CI)	*P*-value	Explained variance (%)
*Organization and coordination of care*						
QI14 Sending of unfixed biopsy material	321	41	0–91			n.a.
QI15 Integrated reporting of pathology techniques	365	89	35–100			11
Extranodal involvement				2.46 (1.10–5.50)	0.03	
Discussed in pathology panel				5.25 (1.75–15.74)	<0.01	
QI16 Pathology report describes all necessary, predefined characteristics	378	14	0–47			23
Discussed in MTC				2.46 (1.10–5.51)	0.03	
Hospital region 1				2.73 (0.62–12.04)		
2				0.17 (0.02–1.31)		
3				Ref.	0.04	
QI17 Patients discussed in multidisciplinary consultations	422	41	4–96			9
Availability of PET-scanner				4.22 (1.01–17.56)	0.05	
QI18 Results of bone marrow pathology known before start of treatment	317	83	43–100			11
Aggressive tumor				0.30 (0.13–0.70)	<0.01	
Aberrant Hb level				0.38 (0.20–0.72)	<0.01	
QI19 Diagnostic period of 4 weeks after first visit to the hospital	420	47	22–70			10
Previous malignancies				0.52 (0.29–0.95)	0.03	
Extranodal involvement				1.72 (1.11–2.66)	0.02	
In-hospital referral				0.52 (0.34–0.81)	<0.01	
High LDH level				1.9 2 (1.27–3.03)	<0.01	
QI20 Start of therapy within 2 weeks after diagnostic period	313	58	37–79			9
Male gender				1.69 (1.03–2.76)	0.04	
Aggressive tumor				1.99 (1.17–3.41)	0.01	
High LDH level				1.92 (1.16–3.19)	0.01	

Abbreviations: MTC, multidisciplinary team consultation; DLBCL, diffuse large B-cell lymphoma; LDH, lactate-dehydrogenase; Hb, Hemoglobin; RCHOP, ritixumab-involved chemotherapy

In all three domains, QI scores differed considerably between the 19 participating hospitals; variation in guideline adherence (>20 %) among the hospitals was noted in all 20 QIs. The lowest hospital variation was seen for QIs concerning diagnosis of NHL based on morphology and immune phenotype (QI3) and assessment of LDH level (QI6), respectively 26 % (range 74–100) and 30 % (range 70–100). High variation (>50 %) between the hospitals was seen in 12 of the 20 QIs, most frequently (*N* = 5) in the treatment and follow-up domain.

### Determinants of guideline adherence in NHL care

Table 3 displays, per quality indicator, the determinants that significantly influence hospital variation concerning guideline adherence for NHL care. In multilevel modeling, 15 of the 22 characteristics were involved with variation in guideline adherence: 13 at patient level and 2 at hospital level. Several characteristics influenced hospital variation in only 1 quality indicator, including gender, co-morbidities, previous malignancies, referral to another specialist, presence of a pathology panel and PET-scanner.

Determinants associated with 2 to 5 quality indicators were extranodal involvement, MTC, DLBCL tumor type, tumor aggressiveness, LDH and Hb level, therapy used and hospital region. Of these 8 determinants, only therapy showed a clear direction of effect: patients receiving therapy were more likely to receive care as described in the guidelines, including Ann Arbor classification (QI2), performing all staging techniques (QI4) and assessment of IPI (QI5) and LDH level (QI6). The other determinants were both negatively (OR < 1) and positively (OR > 1) linked to guideline adherence. For example, patients discussed in an MTC were less likely to receive an LDH level assessment (QI6), whereas they were more likely to receive all staging techniques (QI4).

The main patient characteristic associated with guideline adherence was age (5 out of 20 quality indicators). In particular, older people were less likely to receive care as measured by the QIs, including correct diagnostic biopsy performance (QI1), Ann Arbor staging (QI2), performing all staging techniques (QI4), assessment of IPI risk factor (QI5), and receiving R-CHOP chemotherapy (QI12), compared to younger patients.

Hospital characteristics associated with QI scores in this dataset included hospital region and an in-hospital PET-scanner. Hospitals in region 2 showed more documentation of target lesions in radiology reports after therapy (QI10) and more complete reports therapy evaluations (QI11), whereas they showed less complete reports for pathology (QI16), compared to the other two regions. The availability of an in-hospital PET-scanner was positively associated with a multidisciplinary discussion of patients (QI17). Participation in trials and hospital size were not included in multivariate analysis, because of the high correlation with hospital type, and availability of an in-hospital pathology laboratory and PET-scanner.

Table 3 also shows the explained variance of the determinants included in the final multilevel model. A substantial part of the variation in guideline adherence can be explained by patient and/or hospital characteristics: ten QIs showed that determinants could explain the variation for at least 10 %. Variation regarding Ann Arbor staging (QI2), evaluation with CT-scans after therapy (QI11) and R-CHOP chemotherapy for DLBCL patients (QI12) showed relatively large explained variances of, respectively, 35, 30 and 26 %.

## Discussion

This study demonstrated substantial hospital variation in guideline adherence for NHL care. Fifteen characteristics at the patient level could partly explain this variation, such as extranodal involvement, multidisciplinary consultation, tumor type, therapy used and hospital region. Hospital characteristics contributed less to the variation in adherence than patient, tumor and professional related characteristics. Patients’ age was involved most frequently as determinant, illustrating that older people are less likely to receive NHL care as described in the guidelines.

Our study showed large gaps between daily practice performance and care as described in the evidence-based guidelines. Large variation in guideline adherence between hospitals is often associated with lower quality of care, since guidelines aim to assist professionals to deliver the most optimal care. However, less adherence does not always indicate lower quality of care: complying with patient preferences or performing less diagnostics due to a low performance status can also point towards patient-centered, safe and deliberately delivered care. It is believed that variation due to deliberately deviate from guidelines is reflected in the upper 10 % of QI scores (90–100 %). Therefore, many studies indicate room for improvement if guideline adherence, as measured by indicators, is below 90 % [9, 22–24]. In our study, 18 out of 20 QIs showed room for improvement, of which 12 QIs demonstrated high hospital variation (>50 %), indicating other factors than patient preferences or performance status might play a role. Similar to our study, Weeks et al. [27] found high variation in NHL management decisions, for example in performing a PET-scan (range 38–95 %) or a bone marrow biopsy (range 21–99 %). Studies concerning other tumor types also showed variation in delivered care between hospitals [18, 28–31].

While this is the first study to investigate determinants at patient as well as hospital level for guideline adherence, and indirectly for the quality of care for NHL patients, other studies examining multilevel determinants have been carried out in several areas, including lung, prostate and (colo)rectal cancer [18, 29, 32–34]. Schroeck et al. [33] provided insight into adherence to QIs for prostate cancer and its regional variation. Most measures showed low adherence rates and high regional variation, for example 72 % variation in follow-up with radiation oncologists (range 14–86 %). They showed that characteristics such as age, clinical stage and number of urologists explained the differences for 5–20 %. Etzioni et al. [32] showed that characteristics as higher-volume surgeons and teaching hospitals contributed to long-term survival in rectal cancer patients, whereas Sacerdote et al. [34] found several social, clinical and hospital characteristics to be associated with the treatment of colorectal cancer, for example, age, gender, hospital volume and an in-hospital radiotherapy service. Mathoulin et al. [29] investigated the quality of colorectal cancer surgery and found several associations with patient, tumor and hospital related factors, such as age, disease stage and hospital type. Finally, Ouwens et al. [18] found patient characteristics to have a greater influence on quality of integrated care than professional or hospital characteristics for patients with non-small cell lung cancer.

Several determinants of guideline adherence and NHL care were found in our study as well. Regarding patient factors, especially patients’ age appeared to influence variation in guideline adherence for NHL care most. For older patients, it can be argued that suboptimal diagnostics and suboptimal but better tolerated therapies sometimes are the best achievable care. However, the reasons for deviation from the guideline should be well thought out and documented by the professionals, which may be influenced by available information for decision making, professionals’ choice or patient preferences. Unfortunately, we were not able to include arguments to deliberately deviate from guideline recommendations, since these are frequently not documented (in a standardized way) in medical records.

Previous studies found patients’ age as an important factor for delivered NHL care: they studied elderly DLBCL patients, defined as patients aged over 60 or 75 years [11, 12, 25]. Younger age and better performance status were associated with receiving CHOP-like chemotherapy. Van de Schans et al. [12] showed age as the only factor associated with receiving less than six cycles of CHOP-like chemotherapy (adjusted for variables as gender and co-morbidity). Concerning overall survival, all three studies concluded that optimal therapy for elderly was associated with better outcomes, after case-mix corrections [11, 12, 25]. After multivariate analyses, Trebouet et al. [35] found also a relation between treatment administration and improved survival in patients over 90 years of age with aggressive NHL. An important drawback of intensive chemotherapy is treatment related toxicity. The elderly are more susceptible to complications, which makes it even more important to accurately select patients for therapy [11]. They stated that elderly are more susceptible to develop complications, which makes it even more important to accurately select patients for therapy. The judgment of professionals must be underscored in this selection process. A possible option to optimize outcomes was proposed by Lin et al. [25]; they opted implementation of tailored interventions to improve the performance status of patients before the start of therapy. In addition, in other fields of oncology lower guideline adherence was seen for elderly as well [34, 36]. Suggested reasons for the lower rates were that elderly patients receive less diagnostics and/or therapy for medical reasons, such as higher burden of co-morbidities [34], or diagnosis of advanced disease stages [36], which was initially seen in our dataset as well (data not explicitly shown). However, co-morbidities and disease stage were included in our analyses and age remained a determinant in the final models.

Besides age, several other tumor and patient related determinants were involved in explaining hospital variation, including previous malignancies, LDH and Hb level, gender, co-morbidity, extranodal involvement, tumor type and tumor aggressiveness. Most of these aspects are common factors measured in NHL research concerning prognostic factors and survival analyses [11, 12, 25, 35]. Unfortunately, this literature shows involvement of the factors with survival in univariate analyses, but not in multivariate analyses. Tumor type and aggressiveness are often not assessed, since studies regularly select only DLBCL or aggressive tumors as subjects of interest [11, 25, 37]. Kuper-Hommel et al. [37, 38] investigated differences in therapy and outcome between patients with nodal and extranodal lymphomas in two large population-based studies. They showed that patients with extranodal lymphomas were less often optimally treated but did not find clear differences in overall survival. In our study, patients with extranodal involvement received less often all required staging techniques and showed more often dose reductions during R-CHOP chemotherapy or reductions without reporting the reason.

Not all determinants found seem directly relevant for clinical practice, such as the influence of the Hb level on QI18: pathology results have to be known before the start of treatment. A possible explanation could be that the urge of starting therapy is higher for patients with a aberrant Hb level and an aggressive tumor. It seems valuable to explore these determinants in other NHL populations.

Of the professional and hospital related determinants for hospital variation in NHL care, treatment is an important factor in relation to better survival, as discussed above. Factors as MTC, hospital region, in-hospital referral, PET-scanner and discussion in a pathology panel are often not taken into account in survival analyses. The possible relation of these factors with overall survival is an interesting issue to address in future research. Hospital region will probably be one of the most challenging determinants, since hospitals cannot move to another geographical region and regional collaborations are embedded, which might be tough to effect change upon. Nevertheless, guideline adherence and quality of care described per region can give valuable insight into regional differences concerning interpretation and rating of the guideline recommendations and provide possible points of interest for improving quality of care.

Strengths of this study are the large study sample (*N* = 423) derived from a population-based cancer registry and the validated guideline-based QIs used for the assessment of variation in guideline adherence for NHL care. These factors contribute to the reliability of our results. Another factor contributing to a reliable dataset is that trained registration employees of the IKNL collected the data independently of the project team. An additional strength of our study is that 2 levels of potential determinants were included, namely patient and hospital level. Multilevel analyses made it possible to include these factors in one regression model per quality indicator.

There are also some limitations that need to be addressed. First, characteristics at the level of professionals were not taken into account, since NHL care is provided by a multidisciplinary team of a hematologist and/or (radiation)oncologist, radiologist, nuclear physician, pathologist and oncology nurse. It was not possible to relate one professional to one patient, which is necessary for inclusion of characteristics at professional level. However, some professional related factors measured at patients level were included in our study, such as patients discussed in MTC and therapy used. Second, only two of eight hospital characteristics included for analyses were found to have significant impact in the final multilevel models. This can be caused by the limited sample size of 19 hospitals, indicating more hospitals may be needed for possible future research. Third, no hospitals from the Western part of the Netherlands were included in our study, which might have introduced some selection bias. However, we did include 19 of the 91 Dutch hospitals, including three different regions, representing 21 % of the Dutch hospital population. Last, a significant amount (>50 %) of data was missing for the parameters performance status and IPI score. One of the reasons for this could be that only official WHO scores and Karnofsky scores were collected, excluding general terms as ‘healthy man’ or ‘vital women’. Arguments for not calculating the IPI score included that therapy choices do not change for most patients based on the IPI score, except for patients participating in clinical trials.

## Conclusion

In conclusion, this study showed considerable hospital variation in guideline adherence, as an indication for quality of delivered NHL care, including the domains diagnosis and staging, treatment and follow-up and organization and coordination of care. Our study demonstrated that patient characteristics appear to have more influence on guideline adherence than hospital characteristics, especially patients’ age. Tailored strategies to optimize NHL care should take into account the determinants identified in this study. Especially for older patients, reasons for not performing all necessary diagnostics and staging techniques should be a topic of interest, taking into account safe and patient-centered care as well.

## Abbreviations

CMO: Research ethics committee
cRCT: Cluster randomized controlled trial
DLBCL: Diffuse large B-cell lymphoma
Hb: Hemoglobin
IKNL: Netherlands Comprehensive Cancer Organisation
IOM: Institute of Medicine
IPI: International prognostic index
LDH: Lactate-dehydrogenase
MTC: Multidisciplinary team consultation
NHL: Non-Hodgkin’s lymphoma
OR: Odds ratio
PEARL: Improvement of patiEnts’ hospitAl caRe for non-hodgkin’s Lymphoma
PET: Positron emission tomography
QIs: Quality indicators
R-CHOP chemotherapy: Chemotherapy regimen consisting of Rituximab, Cyclophosphamide, Doxorubicin hydrochloride, Vincristine (Oncovin) and Prednisone.
WHO: World Health Organization

## References

[1] Bray F, Jemal A, Grey N, Ferlay J, Forman D (2012). Global cancer transitions according to the Human Development Index (2008–2030): a population-based study. Lancet Oncol.

[2] Siegel R, Naishadham D, Jemal A (2013). Cancer statistics, 2013. CA Cancer J Clin.

[3] Lu P (2005). Staging and classification of lymphoma. Semin Nucl Med.

[4] Sant M, Minicozzi P, Mounier M, Anderson LA, Brenner H, Holleczek B (2014). Survival for haematological malignancies in Europe between 1997 and 2008 by region and age: results of EUROCARE-5, a population-based study. Lancet Oncol.

[5] Howlader N, Noone AM, Krapcho M, Garshell J, Miller D, Altekruse SF (2014). SEER Cancer Statistics Review 1975–2011. National Cancer Institute.

[6] IOM (2001). Crossing the Quality Chasm: A New Health System for the 21st Century.

[7] Berrios-Rivera JP, Fang S, Cabanillas ME, Cabanillas F, Lu H, Du XL (2007). Variations in chemotherapy and radiation therapy in a large nationwide and community-based cohort of elderly patients with non-Hodgkin lymphoma. Am J Clin Oncol.

[8] Friedberg JW, Taylor MD, Cerhan JR, Flowers CR, Dillon H, Farber CM (2009). Follicular lymphoma in the United States: first report of the national LymphoCare study. J Clin Oncol.

[9] Wennekes L, Ottevanger PB, Raemaekers JM, Schouten HC, de Kok MW, Punt CJ (2011). Development and measurement of guideline-based indicators for patients with non-Hodgkin’s lymphoma. J Clin Oncol.

[10] Lawrence M, Olesen F (1997). Indicators of quality in health care. Eur J Gen Pract.

[11] Boslooper K, Kibbelaar R, Storm H, Veeger NJ, Hovenga S, Woolthuis G (2014). Treatment with rituximab, cyclophosphamide, doxorubicin, vincristine and prednisolone is beneficial but toxic in very elderly patients with diffuse large B-cell lymphoma: a population-based cohort study on treatment, toxicity and outcome. Leuk Lymphoma.

[12] van de Schans SA, Wymenga AN, van Spronsen DJ, Schouten HC, Coebergh JW, Janssen-Heijnen ML (2012). Two sides of the medallion: poor treatment tolerance but better survival by standard chemotherapy in elderly patients with advanced-stage diffuse large B-cell lymphoma. Ann Oncol.

[13] Hermens RP, Haagen EC, Nelen WL, Tepe EM, Akkermans R, Kremer JA (2011). Patient and hospital characteristics associated with variation in guideline adherence in intrauterine insemination care. Int J Qual Health Care.

[14] Peters-Klimm F, Laux G, Campbell S, Muller-Tasch T, Lossnitzer N, Schultz JH (2012). Physician and patient predictors of evidence-based prescribing in heart failure: a multilevel study. PLoS One.

[15] Reeves MJ, Gargano J, Maier KS, Broderick JP, Frankel M, LaBresh KA (2010). Patient-level and hospital-level determinants of the quality of acute stroke care: a multilevel modeling approach. Stroke.

[16] Kasje WN, Denig P, Stewart RE, de Graeff PA, Haaijer-Ruskamp FM (2005). Physician, organisational and patient characteristics explaining the use of angiotensin converting enzyme inhibitors in heart failure treatment: a multilevel study. Eur J Clin Pharmacol.

[17] McCavit TL, Lin H, Zhang S, Ahn C, Quinn CT, Flores G (2011). Hospital volume, hospital teaching status, patient socioeconomic status, and outcomes in patients hospitalized with sickle cell disease. Am J Hematol.

[18] Ouwens MM, Hermens RR, Termeer RA, Vonk-Okhuijsen SY, Tjan-Heijnen VC, Verhagen AF (2007). Quality of integrated care for patients with nonsmall cell lung cancer: variations and determinants of care. Cancer.

[19] Trinh QD, Sammon J, Jhaveri J, Sun M, Ghani KR, Schmitges J (2012). Variations in the quality of care at radical prostatectomy. Ther Adv Urol.

[20] Stienen JJ, Ottevanger PB, Wennekes L, van de Schans SA, Dekker HM, van der Maazen RW (2015). Trends in quality of non-Hodgkin’s lymphoma care: is it getting better?. Annals of hematology.

[21] Stienen JJ, Hermens RP, Wennekes L, van de Schans SA, Dekker HM, Blijlevens NM (2013). Improvement of hospital care for patients with non-Hodgkin’s lymphoma: protocol for a cluster randomized controlled trial (PEARL study). IS.

[22] Mourad SM, Nelen WL, Hermens RP, Bancsi LF, Braat DD, Zielhuis GA (2008). Variation in subfertility care measured by guideline-based performance indicators. Hum Reprod.

[23] Ouwens MM, Marres HA, Hermens RR, Hulscher MM, van den Hoogen FJ, Grol RP (2007). Quality of integrated care for patients with head and neck cancer: Development and measurement of clinical indicators. Head Neck.

[24] Smit M, Chan KL, Middeldorp JM, van Roosmalen J (2014). Postpartum haemorrhage in midwifery care in the Netherlands: validation of quality indicators for midwifery guidelines. BMC Pregnancy Childbirth.

[25] Lin TL, Kuo MC, Shih LY, Dunn P, Wang PN, Wu JH (2012). The impact of age, Charlson comorbidity index, and performance status on treatment of elderly patients with diffuse large B cell lymphoma. Ann Hematol.

[26] Leyland AH, Groenewegen PP (2003). Multilevel modelling and public health policy. Scand J Public Health.

[27] Weeks JC, Uno H, Taback N, Ting G, Cronin A, D’Amico TA (2014). Interinstitutional variation in management decisions for treatment of 4 common types of cancer: A multi-institutional cohort study. Ann Intern Med.

[28] Elferink MA, Wouters MW, Krijnen P, Lemmens VE, Jansen-Landheer ML, van de Velde CJ (2010). Disparities in quality of care for colon cancer between hospitals in the Netherlands. Eur J Surg Oncol.

[29] Mathoulin-Pelissier S, Becouarn Y, Belleannee G, Pinon E, Jaffre A, Coureau G (2012). Regional Aquitaine Group for Colorectal cancer G: Quality indicators for colorectal cancer surgery and care according to patient-, tumor-, and hospital-related factors. BMC Cancer.

[30] McCahill LE, Single RM, Aiello Bowles EJ, Feigelson HS, James TA, Barney T (2012). Variability in reexcision following breast conservation surgery. JAMA.

[31] van Leersum NJ, Snijders HS, Wouters MW, Henneman D, Marijnen CA, Rutten HR (2013). Dutch Surgical Colorectal Cancer Audit G: Evaluating national practice of preoperative radiotherapy for rectal cancer based on clinical auditing. Eur J Surg Oncol.

[32] Etzioni DA, Young-Fadok TM, Cima RR, Wasif N, Madoff RD, Naessens JM (2014). Patient survival after surgical treatment of rectal cancer: impact of surgeon and hospital characteristics. Cancer.

[33] Schroeck FR, Kaufman SR, Jacobs BL, Skolarus TA, Hollingsworth JM, Shahinian VB (2014). Regional variation in quality of prostate cancer care. J Urol.

[34] Sacerdote C, Baldi I, Bertetto O, Dicuonzo D, Farina E, Pagano E (2012). Hospital factors and patient characteristics in the treatment of colorectal cancer: a population based study. BMC Public Health.

[35] Trebouet A, Marchand T, Lemal R, Gyan E, Broussais-Guillaumot F, Guillermin Y (2013). Lymphoma occurring in patients over 90 years of age: characteristics, outcomes, and prognostic factors. A retrospective analysis of 234 cases from the LYSA. Ann Oncol.

[36] Aparicio T, Navazesh A, Boutron I, Bouarioua N, Chosidow D, Mion M (2009). Half of elderly patients routinely treated for colorectal cancer receive a sub-standard treatment. Crit Rev Oncol Hematol.

[37] Kuper-Hommel MJ, van de Schans SA, Vreugdenhil G, van Krieken JH, Coebergh JW (2013). Undertreatment of patients with localized extranodal compared with nodal diffuse large B-cell lymphoma. Leuk Lymphoma.

[38] Kuper-Hommel MJ, van de Schans SA, Vreugdenhil G, van Krieken JH, Coebergh JW (2013). Trends in incidence, therapy and outcome of localized nodal and extranodal marginal zone lymphomas: declining incidence and inferior outcome for gastrointestinal sites. Leuk Lymphoma.

